# New evidence of trends in cognitive function among middle-aged and older adults in China, 2011-2018: an age-period-cohort analysis

**DOI:** 10.1186/s12877-023-04166-9

**Published:** 2023-08-21

**Authors:** Shuai Guo, Xiao-Ying Zheng

**Affiliations:** School of Population Medicine and Public Health, Chinese Academy of Medical Sciences & Peking Union Medical College, No. 31, Road 3rd, Bei-Ji-Ge, Dongcheng District, Beijing, 100073 P.R. China

**Keywords:** Cognitive function, Age-period-cohort analysis, China

## Abstract

**Background:**

Increasing evidence from high-income countries suggests the risk of cognitive impairment has been declining recently. However, related studies in China have rarely been done, and the results are inconsistent. We analyze the trends in cognitive function among middle-aged and older adults in China between 2011 and 2018.

**Methods:**

We used data from four waves of the China Health and Retirement Longitudinal Study (CHARLS), including 48918 individuals aged 45 years and older. Cognitive function was assessed using the CHARLS cognitive measures containing episodic memory, orientation, attention, and visuospatial abilities. The hierarchical age-period-cohort (APC) model was used to quantify the separate age, period, and cohort effects on trends in cognitive function.

**Results:**

The study sample’s ages ranged from 45 to 105 years (Mean = 59.2, SD = 9.4). Cognitive function declined with age net of period and cohort effects, an apparent acceleration in the rate of cognitive decline after age 65 was found adjusting for individual characteristics. Although period effects on trends in cognitive function remained stable during the study period, hierarchical APC models demonstrated significant cohort variations. Independent of age and period effects, there was a fluctuating trend across cohorts before 1960 and an overall decline across successive cohorts.

**Conclusions:**

Our study indicates that the age effect remains the most crucial factor regarding cognitive decline. Moreover, results demonstrate that cohorts living in social upheaval leading to educational deprivation and/or nutritional deficiency in early life may face a higher risk for cognitive deterioration later in life. Such findings indicate that dementia prevention from a life course perspective and cohort-specific strategies are critical to alleviating the future public-health burdens related to cognitive aging. Ongoing attention should be paid to the role of cross-cohort differences in education on cohort trends in cognition in countries like China that are aging rapidly and have a late start in educational expansion compared to other countries. Other factors, such as environmental stimulation, need to be noticed in younger cohorts.

**Supplementary Information:**

The online version contains supplementary material available at 10.1186/s12877-023-04166-9.

## Introduction

The increase in life expectancy has led to many studies on age-related pathologies such as cognitive impairment, including dementia. Along with the global trend of population aging, the number of people living with dementia may be rising in parallel [1]. In China, demographic and epidemiological transitions have contributed to the rise in the socioeconomic burden of dementia since the late 20th century [2]. In 2016, the number of patients with dementia in China accounted for about 25% of the global population with dementia. Moreover, compared with an increase of 1.7% in the age-standardized dementia prevalence worldwide from 1990 to 2016, the prevalence in China increased by 5.6% [3]. Therefore, more understanding of trends in cognitive function is needed for the improvement of population health and the development of health policy.

In West Europe and North America, consistent favorable trends were found among older adults in terms of dementia [4, 5], cognitive impairment [6, 7], and cognitive performance [8]. The major mechanisms for such secular trends are the increase in education attainment, improvements in living conditions, and developments for the treatment of cardiovascular disease [5, 7]. Meanwhile, related studies in Asian countries have not verified the pattern of secular trends [9, 10]. In China, research on trends in cognitive function has rarely been done owing to a lack of comparable data [5]. Previous systematic analysis and meta-analysis suggest that the temporal variation of dementia prevalence in older Chinese shows a fluctuating or upward trend [11, 12]. However, when considering cognitive test performance and cognitive impairment, existing studies mainly conclude that cognitive function among older adults has not deteriorated over time — researchers found that the cognitive ability remained stable over time [13, 14]; in addition, decreasing trends were found in the prevalence and incidence of cognitive impairment controlling for the age structure [15, 16]. Given the mixed result on trends in cognitive function of the Chinese population, it is necessary to explore this issue more in-depth.

**Fig. 1:**
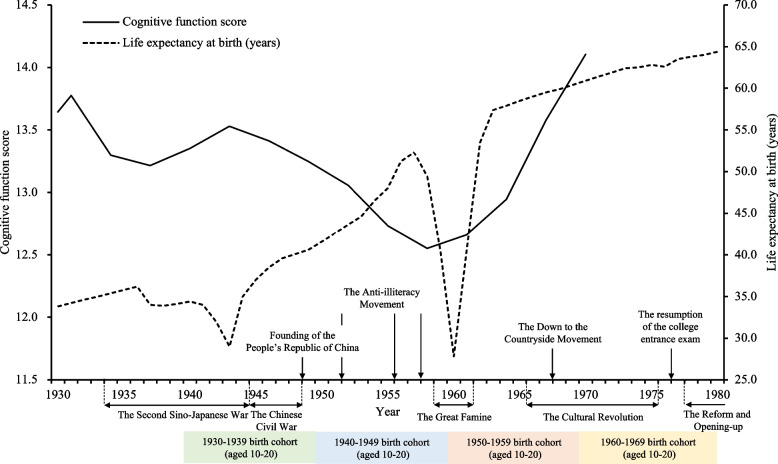
Predicted cognitive function score during the study period, life expectancy at birth, and relevant historical events between 1930 and 1980. The dotted line in the figure represents the predicted cognitive function score based on Model 4; the solid line represents life expectancy in each year, which was obtained from Gapminder

Understanding the cohort differences in the mean level of cognition and cognitive aging will provide important clues to explore the development of cognitive health at the population level. Throughout the 20th century, it has been noted that younger cohorts consistently surpassed earlier generations on IQ tests, known as the Flynn Effect [17]. Based on longitudinal data, Schaie and colleagues found improved cohort trends in crystallized and fluid intelligence and a lower decline rate in age-related changes even before the 1990s [18, 19]. The Flynn Effect was found in high-income countries (HICs) other than the United States in subsequent studies [20–22], and there is evidence that this effect may maintain in very older people (> 90 years) [23], rather than counteracted by mortality-related cognitive decline [19, 24]. However, in limited studies about cohort differences in cognition among older Chinese adults, while evidence exists for the Flynn Effect [14], there is more research finding that cognitive function deteriorated across successive cohorts among those born in the first half of the 20th century [25, 26]. Researchers suggest that lower educational attainment and childhood adversity resulting from frequent warfare during this period may explain such cross-cohort differences. As shown in Fig. 1, the Second Sino-Japanese War caused a dramatic decline in life expectancy of the Chinese population. In conclusion, adding new evidence on cohort differences in cognitive function among older adults in China is crucial.

Previous studies in China rarely controlled for three time-related changes to health trends simultaneously, which are age, period, and cohort effects. Intuitively, aging effects reflect biological and social processes of cognitive aging internal to individuals, period effects represent variation in cognitive function influencing the whole population over time, and cohort effects subsume changes in cognitive function across successive generations [27]. Although some studies have examined the age-period-cohort (APC) effects on trends in cognitive function in Chinese adults, they focused on populations aged 65 or older [13, 28]. Therefore, there is insufficient knowledge of cognitive function among those born after the 1950s. Is it possible to detect the Flynn Effect after the founding of the new China — a period of social stability (As Fig. 1 points out, after the founding of the People’s Republic of China, life expectancy has generally increased steadily and rapidly, except for a sharp decline during the Great Famine)? This is an open question that deserves to be explored. Moreover, from a life course perspective, dementia prevention requires attention to cognitive function and its impact factors in the middle-aged population [1].

To further address these questions, we used data from the China Health and Retirement Longitudinal Study (CHARLS), a nationally representative longitudinal survey, to quantify APC effects on trends in cognitive function among Chinese adults aged 45 years and older from 2011 to 2018.

## Materials and methods

### Data and study sample

The CHARLS is a longitudinal survey starting in 2011 with three following waves in 2013, 2015, and 2018, colleting socio-demographic information, socioeconomics status, health status, and related factors. A stratified multi-stage probability proportional to size sampling method was used to obtain a nationally representative sample of Chinses residents aged 45 years and older in 450 villages/urban communities, 150 countries/districts, and 28 provinces. To ensure the population representativeness as the study sample aged, refreshment samples have been added to each following wave [29].

**Fig. 2:**
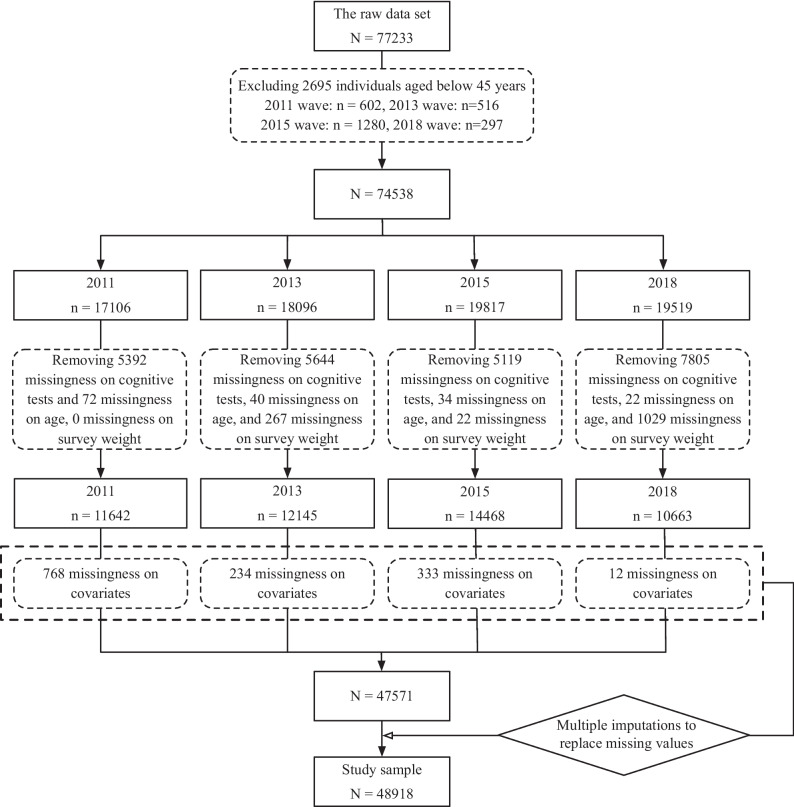
Flowchart of the study sample

As shown in Fig. 2, the raw CHARLS data set contains 77233 respondents. After excluding those under 45 years old (*n* = 2695, resulting in 74538 observations), observations with missing information on cognitive tests (missingness on any item in the cognitive tests, *n* = 23960), age (*n* = 168), and survey weight (*n* = 1492) were excluded, resulting in 48918 individuals (11642, 12145, 14468, and 10663 in 2011, 2013, 2015, and 2018, respectively) as the study sample. Among 48918 individuals, 47571 individuals had no missing value on covariates. Multiple imputations were performed to impute missing values of all covariates. Details on the distribution of missing values in the study sample and operation details of multiple imputations can be found in the Statistical analyses section.

Considering the high proportion of missingness on cognitive tests, we described the number and types of missing values for each dimension of cognitive function by survey waves. As shown in Table S1 (in Additional file 1), except for the 2013 survey, the percentage of missing values due to “missing responses” was generally not high. The high percentage of missingness due to “don’t know” in the orientation and attention dimensions may reflect the overall low education level of the middle-aged and older adults interviewed. Moreover, due to the study design, some respondents were not assessed in the 2018 survey (962 in the orientation dimension and 2572 in the visuospatial ability dimension). However, the small number of missing values due to “not assessed” is unlikely to bias the results systematically.

### Measurement of cognitive function

Four dimensions of cognitive function were assessed in CHARLS: episodic memory, orientation, attention, and visuospatial abilities. Memory was assessed by word recall. Respondents were asked to immediately repeat as many words as they could in any order after interviews read a list containing 10 Chinese nouns (e.g., “Shore”, “Letter”, “Arm” etc.) to them (immediate recall). Four minutes later, respondents were asked to recall as many original words as possible (delayed recall) [30]. We formed an episodic memory score as the average number of immediate and delayed recalls based on previous recommendations [31], which ranged from 0 to 10. Orientation and attention were measured by the Telephone Interview of Cognitive Status (TICS) [32]. The questions from the TICS used in CHARLS included the following ten items: recalling today’s date (month, day, year), the day of the week, and season of the year, and serial subtraction of 7 from 100 up to five times. The TICS score was the total number of correct answers (ranging from 0 to 10) [30]. Visuospatial abilities were assessed by figure drawing. Respondents were shown a picture of two overlapping pentagons and asked to redraw it. Respondents received a score of 1 if they successfully finished the task, for those who failed received a score of 0 [33]. Consistent with prior CHARLS publications, the sum of all three above measures represented the respondent’s cognitive status (ranging from 0 to 21, Cronbach’s α = 0.835), and higher scores indicated better cognitive functioning [34–37].

### Independent variables

Two sets of variables were included in our analyses: individual and contextual variables.

The key individual variable was age, i.e., the respondent’s age at the time of the survey, which was treated as a continuous variable. It was transformed by centering at the grand mean (59.2 years) and further being divided by 10 for mathematical convenience. Other individual variables were divided into three broad categories — socio-demographic characteristics, socioeconomic status (SES), and health behaviors and health status.

Socio-demographic characteristics included gender, marital status (married with spouse/cohabited, divorced/separated/widowed/single), household registration (urban, rural), and region (west, central, east). SES was assessed by the current work status (not currently working, agricultural job, nonagricultural job) [38] and education (illiterate, <middle school, ≥middle school). Health behaviors and health status were measured through drinking status last year (heavy drinkers: drank more than/equal to twice a day; mild/moderate drinkers: drank less than twice a day; nondrinkers: never had any alcoholic beverage) [35], smoking status (heavy smokers: ≥20 cigarettes per day currently; mild/moderate smokers: <20 cigarettes per day currently or quit smoking; nonsmokers: never smoked) [35], participation in social activities (yes: participated in any of the following six activities last month — interacting with friends; playing Ma-jong, playing chess, playing cards, or going to the community club; going to a sport, social, or other kind of club; taking part in a community-related organization; doing voluntary or charity work; and attending an educational or training course), and self-rated health status (continuous variable ranged from “very poor = 1” to “very good = 5” on a 5-point scale).

The period and birth cohort were regarded as contextual variables. Specifically, the period was determined by survey years from 2011 to 2018 and generated as a four-category variable. The birth cohort was determined by three-year cohorts ranging from 1910 to 1973. To ensure an adequate sample size, the oldest cohort included twenty-one years (from 1910 to 1930), and the youngest included four years (from 1970 to 1973). As a result, respondents’ birth cohorts were divided into 15 groups, specifically: 1910-1930, 1931-1933, 1934-1936, 1937-1939, 1940-1942, 1943-1945, 1946-1948, 1949-1951, 1952-1954, 1955-1957, 1958-1960, 1961-1963, 1964-1966, 1967-1969, and 1970-1973. The distribution of the study sample cross-classified by period and cohort is shown in Table 1.

**Table 1:** Sample size distribution by survey year and birth cohort

	2011		2013		2015		2018		Total	
Cohort	n	%	n	%	n	%	n	%	N	%
1910-1930	157	1.35	92	0.76	62	0.43	15	0.14	326	0.67
1931-1933	184	1.58	123	1.01	100	0.69	31	0.29	438	0.90
1934-1936	304	2.61	247	2.03	228	1.58	82	0.77	861	1.76
1937-1939	427	3.67	374	3.08	371	2.56	175	1.64	1347	2.75
1940-1942	615	5.28	533	4.39	587	4.06	335	3.14	2070	4.23
1943-1945	741	6.36	702	5.78	745	5.15	464	4.35	2652	5.42
1946-1948	1021	8.77	989	8.14	1035	7.15	684	6.41	3729	7.62
1949-1951	1265	10.87	1250	10.29	1378	9.52	961	9.01	4854	9.92
1952-1954	1494	12.83	1451	11.95	1594	11.02	1083	10.16	5622	11.49
1955-1957	1472	12.64	1474	12.14	1629	11.26	1184	11.10	5759	11.77
1958-1960	1141	9.80	1184	9.75	1304	9.01	1016	9.53	4645	9.50
1961-1963	1364	11.72	1406	11.58	1565	10.82	1194	11.20	5529	11.30
1964-1966	1454	12.49	1645	13.54	1933	13.36	1519	14.25	6551	13.39
1967-1969	3	0.03	675	5.56	1510	10.44	1186	11.12	3374	6.90
1970-1973	0	0.00	0	0.00	427	2.95	734	6.88	1161	2.37
Total	11642	100	12145	100	14468	100	10663	100	48918	100

### Statistical analyses

Our main analyses begin with a descriptive analysis of the study variables for each survey and then postulate the cross-classified random modeling (CCREM) specification of the hierarchical APC models proposed by Yang and Land [39]. The CCREM is a two-level model — individual characteristics are level-1 variables, and period and cohort are level-2 variables. This model setup allows individuals to be nested within “cells” created by cross-classifying two kinds of contextual variables: surveys and birth cohorts. Such a model may be expressed as follows:

1 $${Cog}_{ijk}={\beta }_{0jk}+{\beta }_{1}{Age}_{ijk}+{\beta }_{2}{Age}_{ijk}^{2}+\sum_{p=3}^{P}{\beta }_{p}{X}_{p}+{e}_{ijk}$$

where ${Cog}_{ijk}$ is the measure of cognitive function of the respondent $i$ for $i=1, \dots , {n}_{jk}$ individuals within period $j$ ($j=1, \dots , 4$) and cohort $k$ ($k=1, \dots , 15$). ${Age}_{ijk}$ and ${Age}_{ijk}^{2}$ represent age and age-squared, respectively; $X$ denotes the vector of other individual variables. ${\beta }_{1}, \dots , {\beta }_{p}$ are level-1 fixed effects and $P$ is the maximum number of independent variables. ${\beta }_{0jk}$ is the intercept indicating the mean cognitive function score for the reference group at mean age nested in period $j$ and cohort $k$. ${e}_{ijk}$ is the random individual effect.

Random intercepts embedded in Eq. 1 may be expressed as follows:

2 $${\beta }_{0jk}={\gamma }_{0}+{\mu }_{0j}+{\nu }_{0k}$$

where ${\gamma }_{0}$ is the grand-mean cognitive function score of all respondents at mean age from the reference group, overall periods and cohorts. ${\mu }_{0j}$ and ${\nu }_{0k}$ donate period and cohort effects, respectively, and estimated as level-2 residual random effects. Specifically, ${\mu }_{0j}$ is the contribution of period $j$ on ${\beta }_{0jk}$ averaged over all cohorts. Similarly, ${\nu }_{0k}$ is the contribution of cohort $k$ on ${\beta }_{0jk}$ averaged over all periods. The CCREM can be estimated based on the combined model in Eqs. 1 and 2. Linear CCREMs were calculated using SAS PROC GLIMMIX [40]. In all analyses, CHARLS individual weights were used to adjust for complex sampling design and non-response of the survey [29].

To reduce the potential bias of missing values and maximize the utility of the available data, we conducted the analysis using the method of multiple imputation by chained equations [41]. Firstly, the number and percentage of missing observations for each variable by survey wave were summarized (see Table S2 in Additional file 1). Secondly, Little’s chi-squared test of missing completely at random (MCAR) and covariate-dependent missingness (CDM) was implemented to analyze the missing-value mechanism [42]. The MCAR test proves that the missing data are not MCAR under significance level 0.05 (${\chi }^{2}$ distance = 180.1, Degrees of freedom = 30, *P* <.001). After adding age, gender, marital status, household registration, household registration, and education as covariates into the test, the CDM test implies that the missing-data mechanism can be reasonably regarded as CDM given the covariates (${\chi }^{2}$ distance = 279.7, Degrees of freedom = 390, *P* = 1.000). Therefore, implementing multiple imputations is reasonable as the MCAR assumption is violated. Finally, we replaced missing data with the mean of the five imputed values for CCREMs [43]. All of the above analyses were conducted in STATA 16.0.

## Results

### Characteristics of the study sample

Table 2 shows the descriptive results of the study sample from 2011 to 2018. The mean cognitive function score remained stable overall, but the sample in 2018 had a significantly higher score $\left(P < .001, {\eta }^{2}= .006\right)$. Regarding the socio-demographic characteristics, although the sample became older $\left(P < .001,{\eta }^{2}= .008\right)$ and more of them lost their spouses $\left(P < .001,{\phi }_{C}= .020\right)$, the distributions of household registration and regions did not change significantly over time. The SES improved during the study period; substantial declines were found in the percent of agricultural jobs $\left(31.6\mathrm{\% in }2011\mathrm{ vs}. 27.4\mathrm{\% in }2018;P< .001, {\phi }_{C}= .073\right)$ and illiterate $\left(18.0\mathrm{\% in }2011\mathrm{ vs}. 8.0\mathrm{\% in }2018; P < .001, {\phi }_{C}=.103\right)$. Considering the health behaviors and health status, the results indicated an increasing proportion of those who lived an unhealthy lifestyle. Nondrinkers comprised 67.6% of the sample in 2011 but 58.4% in 2018 $\left(P < .001,{\phi }_{C}=.056\right)$. Similarly, the proportion of nonsmokers declined monotonically from 58.9% in 2011 to 53.7% in 2018 $\left(P < .001,{\phi }_{C}=.107\right)$. Though the proportions of those who participated in social activities were higher in the last three survey waves, a declining trend was also found, from 59.1% to 52.3% $\left(P < .001,{\phi }_{C}=.059\right)$.

**Table 2:** Characteristics of the CHARLS analytical sample by survey wave

	2011	2013	2015	2018	Total		
Characteristic	*n* = 11642	*n* = 12145	*n* = 14468	*n* = 10633	*N* = 48918	*P*^a^	Effect size^b^
Cognition score (0-21)						<.001	0.006
Mean (SD)	12.5 (3.3)	12.7 (3.3)	12.5 (3.4)	**13.0 (3.4)**	12.7 (3.4)		
Age, years (45-105)						<.001	0.008
Mean (SD)	58.4 (9.3)	58.8 (9.3)	59.2 (9.8)	**60.6 (8.9)**	59.2 (9.4)		
Gender						0.010	0.015
Men	**6053 (51.3)**	6349 (52.4)	7694 (53.4)	5758 (54.2)	25854 (52.9)		
Women	**5589 (48.7)**	5796 (47.6)	6774 (46.6)	4905 (45.8)	23064 (47.2)		
Marital status						<.001	0.020
Having spouse	9881 (83.3)	10306 (83.5)	12094 (83.6)	**8867 (83.2)**	41148 (83.4)		
Alone	1761 (16.7)	1839 (16.5)	2374 (16.4)	**1796 (16.8)**	7770 (16.6)		
Household registration						0.544	0.007
Rural	6564 (47.1)	6948 (47.7)	8219 (46.6)	6017 (44.5)	27748 (46.5)		
Urban	5078 (52.9)	5197 (52.3)	6249 (63.4)	4646 (55.5)	21170 (53.5)		
Region						0.118	0.014
West	3531 (28.2)	3710 (28.0)	4490 (28.5)	3225 (28.1)	14956 (28.2)		
Central	3939 (31.5)	4068 (31.2)	4770 (31.1)	3710 (27.4)	16487 (31.7)		
East	4172 (40.3)	4367 (40.8)	5208 (40.4)	3728 (34.5)	17475 (40.1)		
Occupation						<.001	0.073
Nonworking	**4129 (39.5)**	3863 (35.9)	4592 (35.2)	3443 (38.1)	16027 (37.1)		
Agricultural job	**4401 (31.6)**	4730 (33.2)	4943 (28.6)	3589 (27.4)	17663 (30.2)		
Nonagricultural job	**3112 (28.9)**	3552 (30.9)	4933 (36.2)	3631 (34.5)	15228 (32.7)		
Education						<.001	0.103
Illiterate	2254 (18.0)	1942 (14.4)	2210 (13.3)	**1004 (8.0)**	7410 (13.5)		
<middle school	4803 (39.1)	5176 (40.3)	6855 (46.0)	**5172 (46.4)**	22006 (43.0)		
≥middle school	4585 (42.9)	5027 (45.2)	5403 (40.8)	**4487 (45.7)**	19502 (43.5)		
Drinking						<.001	0.056
Nondrinker	**7851 (67.6)**	7546 (61.8)	8903 (60.8)	6468 (58.4)	30768 (62.1)		
Light/moderate	**3622 (31.2)**	4387 (36.7)	5347 (37.7)	4060 (40.5)	17416 (36.6)		
Heavy	**169 (1.3)**	212 (1.5)	218 (1.5)	135 (1.1)	734 (1.3)		
Smoking						<.001	0.107
Nonsmoker	**6722 (58.9)**	6703 (56.2)	7652 (53.9)	5670 (53.7)	26747 (55.6)		
Light/moderate	**3150 (26.4)**	4389 (34.6)	4355 (30.2)	3448 (32.6)	15342 (30.9)		
Heavy	**1770 (14.6)**	1053 (9.3)	2461 (15.9)	1545 (13.7)	6829 (13.4)		
Social activities						<.001	0.059
Participating	5750 (50.4)	**6939 (59.1)**	7558 (53.5)	5370 (52.3)	25617 (53.9)		
Not participating	5892 (49.6)	**5206 (40.9)**	6910 (46.5)	5293 (46.5)	23301 (46.1)		
SRH (1-5)						<.001	0.002
Mean (SD)	**3.08 (0.88)**	3.11 (0.91)	3.16 (0.99)	3.15 (0.96)	3.13 (0.94)		

Values are *n* (%) unless otherwise noted, values in parentheses are weighted percentages calculated using the CHARLS sampling weights; *CHARLS* China Health and Retirement Longitudinal Study, *SRH* Self-rated health

^a^The reported *P* value is for a ${\chi }^{2}$ test or One-Way ANOVA for significant differences in proportion or mean between years after adjusting for age and gender differences across survey waves, values in bold font indicate the proportion or mean of a specific variable in this survey wave is significantly different from other waves

^b^The effect size is ${\phi }_{c}$ (Cramér’s V) for ${\chi }^{2}$ test and ${\eta }^{2}$ for ANOVA

### APC effects on cognitive function changings

Table 3 reports estimates of fixed effects coefficients and random-effects variance components from the linear CCREMs. Four CCREMs were built in this study: Model 1 only included age and age-squared in level-1 to test the unadjusted APC effects. From Models 2 to 4, socio-demographic characteristics, SES indicators, health behaviors, and health status were added sequentially to examine whether individual factors might modify the APC effects. Predicted cognitive function scores are estimated and displayed in figures from selected models to illustrate key findings. Bayesian Information Criterion (BIC) statistics were used to compare the goodness of fit between models. Model 4 has a better model fit than previous models, as indicated by the smallest BIC statistic.

**Table 3:** Estimates from linear CCREMs of cognitive function (*N* = 48918)

Fixed effects	Model 1	Model 2	Model 3	Model 4
	β (95% CI)	β (95% CI)	β (95% CI)	β (95% CI)
Intercept	12.91 (12.57,13.25)	12.24 (11.91,12.57)	14.13 (13.81,14.45)	13.27 (12.96,13.59)
Age/10	-0.96 (-1.09,-0.83)	-0.99 (-1.12,-0.86)	-0.64 (-0.80,-0.47)	-0.59 (-0.74,-0.44)
Age^2^/100	-0.16 (-0.22,-0.11)	-0.17 (-0.22,-0.11)	-0.26 (-0.31,-0.21)	-0.25 (-0.30,-0.20)
Female	/	-0.77 (-0.82,-0.71)	-0.05 (-0.08,-0.01)	-0.24 (-0.31,-0.16)
Having spouse	/	0.50 (0.42,0.57)	0.38 (0.31,0.44)	0.37 (0.30,0.44)
Urban	/	1.65 (1.60,1.71)	0.82 (0.76,0.87)	0.72 (0.66,0.78)
Region	/			
East		1(Reference)	1(Reference)	1(Reference)
Central		-0.17 (-0.24,-0.11)	-0.27 (-0.33,-0.21)	-0.23 (-0.29,-0.18)
West		-0.67 (-0.73,-0.60)	-0.56 (-0.63,-0.50)	-0.50 (-0.56,-0.43)
Education	/	/		
≥middle school			1(Reference)	1(Reference)
<middle school			-1.57 (-1.63,-1.52)	-1.49 (-1.55,-1.43)
Illiterate			-4.30 (-4.38,-4.21)	-4.20 (-4.28,-4.11)
Occupation	/	/		
Nonagricultural job			1(Reference)	1(Reference)
Nonworking			-0.17 (-0.24,-0.10)	-0.10 (-0.17,-0.04)
Agricultural job			-0.51 (-0.58,-0.44)	-0.46 (-0.52,-0.39)
Drinking	/	/	/	
Nondrinker				-0.12 (-0.17,-0.06)
Light/moderate				1(Reference)
Heavy				-0.66 (-0.87,-0.44)
Smoking	/	/	/	
Nonsmoker				1(Reference)
Light/moderate				-0.28 (-0.35,-0.20)
Heavy				-0.49 (-0.58,-0.40)
Participating in social activities	/	/	/	0.58 (0.53,0.63)
SRH	/	/	/	0.24 (0.21,0.27)
Random effects (variance components)	σ (95% CI)	σ (95% CI)	σ (95% CI)	σ (95% CI)
Period	0.084 (-0.053,0.221)	0.072 (-0.045,0.189)	0.016 (-0.011,0.043)	0.015 (-0.010,0.040)
Cohort	0.099 (0.015,0.183)	0.094 (0.014,0.174)	0.255 (0.041,0.469)	0.200 (0.036,0.364)
Individual	9.595 (9.464,9.729)	8.725 (8.604,8.848)	6.991 (6.892,7.092)	6.852 (6.754,7.951)
BIC	261259	256586	247046	246058

Random effect coefficients are omitted in the interest of space, which can be found in Table S3 in Additional file 1, sampling weights were used in the model; CCREMs Cross-classified random effects models, *CHARLS* China Health and Retirement Longitudinal Study, *SRH* Self-rated health, *BIC* Bayesian Information Criterion

**Fig. 3:**
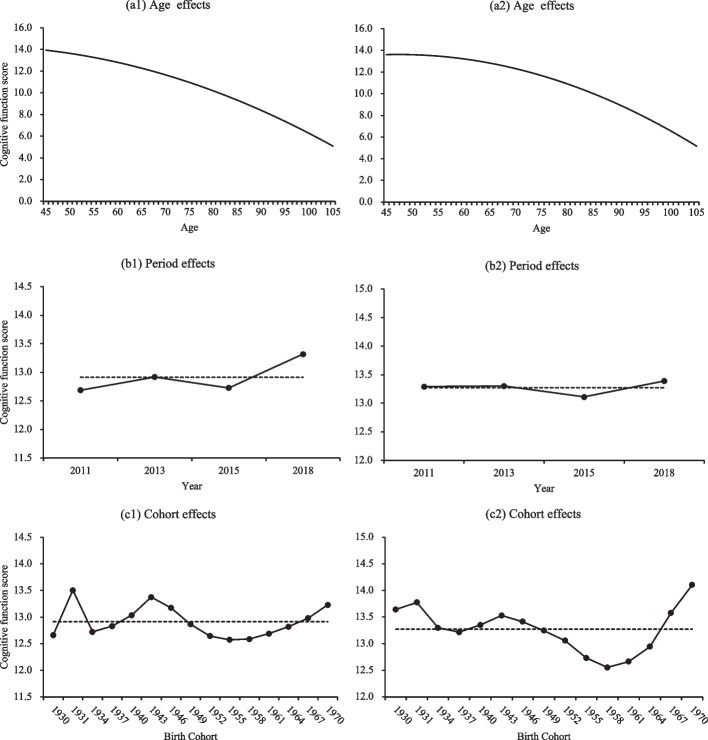
Age, period, and cohort effects on cognitive function. Model 1 (left panel), Model 4 (right panel); the dotted line in the figure represents the estimated grand mean score of cognitive function

The coefficients of age and age squared in Model 1 show a significant quadratic age effect on cognitive function net of period and cohort effects. As shown in Fig. 3(a1), it can be seen that cognitive function declined with advancing age, but the curvilinear shape of the age trajectory is not apparent. The coefficients of age remain significant from Model 2 to Model 4. Moreover, the linear age effect decreased after adjusting for SES in Model 3 compared to Model 2, but the quadratic age effect increased and held when all attributes were considered (Model 4). Figure 3(a2) displays the cognitive function score based on Model 4. Compared with the results from Model 1, we can find a relatively slow rate of cognitive decline before entering old age and an apparent accelerated cognitive decline after age 65.

The estimates of level-2 random effects in terms of variance components in Model 1 indicate significant cohort effects (σ, 0.099; 95% CI, 0.015–0.183), whereas the variation by periods is smaller and not statistically significant (σ, 0.084; 95% CI, -0.053–0.221). Estimated variance components from Models 2 to 4 suggest that, after adjusting for groups of individual characteristics sequentially, the magnitude of period changes in cognitive function was narrowing, but that of cohort changes was expanding. Further considering variance components at the individual level, results in Model 1 show that most of the variance in cognitive function is accounted for by age and age squared, indicating the age effect dominates the APC effects on trends in cognitive function.

Figure 3(b1) and (b2) show the estimated period effects based on Models 1 and 4. The overall pattern of period random effects shows an upward trend net of age and cohort effects. As shown in Fig. 3(b1), the predicted cognitive function score increased from 12.69 to 13.32 between 2011 and 2018. However, after adjusting for all individual characteristics, there are hardly any discernable trends in period effects; the cognitive function score fluctuated between 13.11 and 13.39 during the study period (see Fig. 3(b1)).

The estimated cohort effects based on Models 1 and 4 are shown in Fig. 3(c1) and (c2), respectively. When only APC effects were considered (see Fig. 3(c1)), the pattern of cohort effects suggests that adults born between 1943 and 1955 experienced a monotonous decline in cognitive function. However, after reaching a nadir in 1955, the cognitive function scores gradually rose for subsequent cohorts. For those born before 1943, the cognitive function fluctuated dramatically for cohorts born between 1930 and 1934 and then increased distinctly. When all individual characteristics were controlled (see Fig. 3(c2)), we found the same pattern of cohort trends for those born after 1943 as in Model 1. But more importantly, cognitive function varied more substantially by cohort than in Model 1, especially a solid upward trend was found after 1958. Another interesting finding is an overall decline in cognitive function for cohorts born between 1930 and 1937 rather than the fluctuating trend in Model 1.

Results from Model 2 to Model 4 show that, among the socio-demographic characteristics, men, those married, urban residents, and people living in the eastern region have significantly higher cognitive function scores than their counterparts. However, after adding SES indicators and health behaviors and health status, the associations between all socio-demographic characteristics and cognitive function were attenuated. Regarding the SES indicators, lower educational levels and agricultural jobs were all significant risk factors for cognitive function. Even though controlling for health behaviors and health status, the education effect was substantial and robust. Among health behaviors and health status, non-smoking status, participation in social activities, and higher levels of self-rated health (SRH) was positively related to cognitive function scores. However, light/moderate alcohol consumption was a protective factor for cognitive function.

### Sensitivity analysis

We conducted some robustness checks. Firstly, we used listwise deletion for missingness on covariates (768, 234, 333, and 12 in 2011, 2013, 2015, and 2018, respectively), resulting in a completely observed sample (N = 47571). Then we reran the unadjusted model (Model 1 in Table 3) and full-adjusted model (Model 4 in Table 3) using the completely observed sample. Comparing all estimates in Table 3 with Table S4 in Additional file 1, we found no meaningful differences. In addition, APC effects in terms of predicted cognitive function scores in Figure S1 showed trends similar to that in Fig. 3.

Secondly, we grouped birth cohorts into 5-year intervals (i.e., 1910-1930, 1931-1935, 1936-1940, 1941-1945, 1951-1955, 1956-1960, 1961-1965, 1966-1973), which are conventional in demography [44], and rerun the models. Compared with outcomes from models using 3-year birth groups, similar age and period effects were found in Table S5 and Figure S2 (Additional file 1). The main reason for the observed differences in cohort effects may be that larger time spans smooth out cohort variations in cognitive function after the 5-year cohort group was used. Regardless, overall cohort trends were similar for different model settings.

Thirdly, we regarded responses of “don’t know” (Table S1 in Additional file 1) as incorrect answers and allocated a score of 0. The analytic sample was expanded to 54789 observations (13520, 12145, 17268, and 11856 in 2011, 2013, 2015, and 2018, respectively). As shown in Table S6 and Figure S3 in Additional file 1, estimated results from CCREMs and predicted APC effects were similar compared to the estimates in Table 3 and Fig. 3.

Finally, to verify whether the excluded sample (observations with missingness on cognitive tests/age/survey weight, N = 25620) would cause systematic bias in the study results. We compared the characteristic between excluded and included samples. As shown in Table S7 (Additional file 1), the excluded sample was socioeconomically disadvantaged, especially in terms of education (26.9% in excluded sample vs. 15.2% in included sample; *P* < .001, ${\phi }_{c}$ = .142). However, the distribution of socio-demographic variables was close between the samples, and the effect sizes were small (age: *P* < .001, ${\eta }^{2}$ = .031; gender: *P* < .001, ${\phi }_{c}$ = .043; marriage: *P* < .001, ${\phi }_{c}$ = .036; household registration: *P* = .002, ${\phi }_{c}$ = .011; region: *P* = .004, ${\phi }_{c}$ = .012). Although the proportion of those who participated in social activities was notably lower in the excluded sample (43.4% in excluded sample vs. 52.4% in included sample; *P* < .001, ${\phi }_{c}$ = .039), the proportion of non-drinkers and non-smokers was higher (nondrinker: 70.3% in excluded sample vs. 62.9% in included sample; *P* < .001, ${\phi }_{c}$ = .074; nonsmoker: 58.7% in excluded sample vs. 54.7% in included sample; *P* < .001, ${\phi }_{c}$ = .080). Finally, the SRH levels were similar between the samples (*P* < .001, ${\eta }^{2}$ = .009). In summary, missing values might contribute to an overestimation of cognitive function in the analysis and had a more significant impact on the estimated results of the 2013 survey (notably a higher number of missingness due to “missing responses” than other waves). However, as we focus on the changes in trend, the excluded sample would not bias the interpretation of APC effects.

## Discussion

To our knowledge, this is the first study to use the APC model to analyze trends in cognitive function among middle-aged and older adults for the last seven years in China, based on a nationally representative dataset. Our findings demonstrated age effect is the most apparent effect regarding cognitive decline. Cognitive function decreased with age and showed an accelerated decline after age 65. Although the overall pattern of period effects on cognitive function showed an upward trend from 2011 to 2018, a stable trend was found after controlling for individual characteristics. The lowest cognitive function was found in the 1958-1960 cohort, followed by a significant increase across successive cohorts. Cognitive function remained largely stable for cohorts born before 1943, then declined monotonically until the 1958-1960 cohort.

The results of the CCREMs indicated that APC effects on cognitive function among Chinese adults were distinct and independent of each other. Intuitively, although period effects were minor and statistically insignificant, we found significant and robust age and cohort effects. Therefore, it is of vital importance to estimate separate age, period, and cohort components of change in studies on trends in health [39], especially addressing theoretically relevant concerns in social change and cohort heterogeneity [45].

Regarding the individual characteristics, the APC analysis found evidence supporting the quadratic age effect on cognitive function, consistent with previous studies [13, 19, 46]. Moreover, considering the effect sizes (in terms of predicted values and variance components), the effect of age is noticeably stronger than the effect of period and cohort. This indicates that, with an accompanying continuous decline in mortality and fertility rates, the rapid aging of the population in China will inevitably lead to an increase in the magnitude of older adults with low cognition. However, whether the above phenomenon has severe consequences for social functioning at the population level needs to be explored by more research [47]. After SES was controlled, the linear age effect diminished while the quadratic age effects enhanced. Therefore, the correlation between SES and age-related cognitive decline is not clear. Previous evidence has suggested that education and occupation do not moderate cognitive decline [48–50]. The associations between cognitive function and other individual variables in the present study are mainly in line with previous research findings. An interesting finding is that light/moderate drinking status was a protective factor for cognitive function. Although there is growing evidence that moderate alcohol consumption benefits cognition [51], the complex relationship between alcohol consumption and cognitive function calls for more future research.

After controlling for age and cohort effects, the overall pattern of period effects showed a slight upward trend, but the variation was statistically insignificant. Compared with the results of established studies based on the APC model, Zhang [28] and Hu [13] found that the cognitive function of older Chinese adults remained stable after 2008. The difference in the above results may be due to middle-aged adults being included in this study and different cognition measures being used between studies. In the present study, further consideration of individual characteristics revealed a nearly flat line in cognitive function scores between 2011 and 2018. This indicated that improved SES may have contributed to the upward trend in cognitive function among Chinese adults, as the variance components of period effects decreased by about 80% after adjusting for SES (Model 2 vs. Model 3). The increasing cognitive reserve building from higher educational attainment among more recent cohorts has been one of the main drivers of upward trends in cognitive function in HICs [8, 52]; the present study adds some evidence from China. Moreover, based on the “use it or lose it” hypothesis, people who work in more cognitively demanding jobs have less cognitive decline than those who work in less demanding jobs. In this study, the percentage of those who worked in nonagricultural jobs increased remarkably from 2011 to 2015, followed by stabilization. As more adults work in more cognitively demanding jobs, cognitive function may improve, resulting from better cognitive maintenance at the population level.

The cohort variation in cognitive function found in the present study might indicate the potential influence of societal changes and individual life experiences on cognitive function. As shown in Fig. 1, life expectancy at birth in China is related to the effect of wars (the Second Sino-Japanese War and the Chinese Civil War) and famine (the Great Famine). These historical events, along with other political movements, seem likely to have had a profound effect on living conditions, growth and development, physical and mental health in childhood, and cognition in later life across different generations [5]. The downward trend identified in the 1930-1939 cohort may be mainly due to the prolonged warfare in their teenage years, which reduced their enrollment in primary school [53]. The same cohort trend was confirmed in another study based on data from the Chinese Longitudinal Healthy Longevity Survey in which cognitive function was assessed by Mini-Mental State Examination [25]. For those who were born between 1940 and 1949, cognitive function showed an upward trend and subsequently remained at a relatively high level. We speculate that the Anti-illiteracy Movements implemented during the 1950s might improve the educational opportunities for this cohort [54]. The sharp decline of cognitive function in the 1950-1959 cohort may be caused by the deprivation of further educational opportunities among the “sent-down youths” during the Cultural Revolution [55]. Moreover, prenatal exposure to the Great Famine during 1959-1961 has been proven to have an enduring deleterious effect on mental health later in life [56, 57]. Together with the deterioration of education in their early life [33], this may explain why this birth cohort had the lowest cognitive function. At last, the sharp increase in cognitive function found among those born after 1960 may be due to the stable socioeconomic development after the Cultural Revolution and the resumption of the college entrance exam in 1977. In summary, some evidence of the Flynn Effect can be found in China during a relatively stable social development (e.g., the 1950s, after 1960). On the other hand, when war and social unrest are frequent, the cognitive function of people born or in childhood during this period may be negatively affected through educational deprivation and/or malnutrition. Although cross-cohort differences in education are less powerful in explaining cohort trends in cognitive function in recent studies from HICs [23, 58, 59], an empirical study in China identified the causal effect of education on cognition in elder life even masking the negative impact of nutrition deprivation arising from the Great Famine [33]. This suggests that in countries like China, where rapid aging has occurred in a population with a significantly lower level of education and national income than in HICs, more attention needs to be paid to the effect of education on cognitive function across cohorts; in addition, other factors attributing to the Flynn Effect, such as healthcare, environmental stimulation, and general living conditions should be explored in the younger cohort in China and other low- and middle-income countries (LMICs) [23, 46].

### Limitations

The present study has several limitations. First, we used cognitive assessment scores to evaluate cognitive function rather than clinical evaluations. Therefore, It is not likely that the outcomes will align with the prevalence of cognitive impairment. Moreover, due to data availability, processing speed was not assessed in this analysis, which is an important cognitive domain related to cognitive aging [60]. In wave 4 of CHARLS, the CHARLS-Harmonized Cognitive Assessment Protocol (HCAP) tests were to the survey, which can be used to conduct analyses on Alzheimer’s disease and related dementias [29]. Although the assessment criteria of the CHARLS-HCAP are still under development, we believe this will contribute to future estimates of cognitive impairment and dementia rates in the Chinese population. In 2017, a validation study was conducted to identify the CHARLS-HACP tests from the Health and Retirement Study-HCAP instruments and other tests. The Trail Making Test-Part A was used to measure the information processing speed [61]. However, in the regular wave 4 in 2018, this test was removed from the CHARLS-HACP tests [29]. Therefore, we suggest that the measurement of processing speed should be given attention in future investigations. Second, due to the lack of macro data before the founding of the People’s Republic of China, we did not examine exogenous factors that may explain cohort patterns. Further research should focus on the relationships of macroeconomic variables, cohort characteristics, and cohort changes in cognitive function [62]. Third, the small sample size of the oldest-old respondents resulted in an under-representation of the earliest-born cohort group (1910-1930 cohort), so the cohort effect in this group should be interpreted with caution. Fourth, as noted in the study sample and sensitivity analysis sections, the excluded sample in the present study had significantly lower educational attainment, and this group of people may exhibit unique period or cohort trend in cognitive function. Due to data limitations, we are unable to address this issue effectively. Although we conducted several sensitivity analyses to confirm the robustness of the estimated cohort effects, more high-quality data are needed to provide more accurate estimates of period effects. Lastly, we used data from four waves of the CHARLS between 2011 and 2018 to estimate period effects, which cannot capture long-term and potentially nonlinear change trends in cognitive function. Data gathered over longer periods would be required to provide a more accurate representation of the differences in cognitive function by period.

## Conclusions

Using nationally representative data, we examined the trends in cognitive function among middle-aged and older adults in China based on the APC model. The results suggest that the age effect remains the most crucial factor regarding cognitive decline. However, currently known risk factors account for about 40% of worldwide dementias in the life course. An even greater proportion of dementia is potentially preventable in LMICs [1]. So, there is ample room for dementia prevention and intervention in China. The overall decline in cognitive function found in those born before 1960 may be caused by the deprivation of educational opportunities in early life due to prolonged warfare and social unrest. The present study provides more substantial evidence for the long-term adverse effects of the Great Famine on cognitive function [33, 56], as the net cohort trend was disentangled. Although upward trends in cohort effects were found among those born after 1960, in the context of the rapid educational expansion China experienced since the 1980s [63], whether rising levels of education provide a sustained boost to cognitive function among the more recently born cohorts needs to be explored in subsequent studies. Other LMICs with similar developmental processes as China should also pay attention to the role of cross-cohort differences in educational attainment on the Flynn Effect and should begin to explore other emerging factors found in HICs, like environmental stimulation. Additionally, results suggested that the period effects on cognitive function among Chinese adults remained relatively stable from 2011 to 2018; future research should use more effective methods and a longer data span to analyze period patterns of cognitive function trends in China.

### Supplementary Information

**Additional file 1:** **Table S1.** Summary statistics of missingness in each cognitive domain by survey wave. **Table S2.** Summary statistics of missing data by survey wave. **Table S3.** Estimates of period and cohort effects from linear CCREMs of cognitive function (*N* = 48918). **Table S4.** Estimates from linear CCREMs of cognitive function, completely observed sample (*N* = 47571). **Figure S1.** Age, period, and cohort effects on cognitive function (completely observed sample). Note: Model 1 in Table S3 (left panel), Model 2 in Table S3 (right panel); the dotted line in the figure represents the estimated grand mean score of cognitive function. **Table S5.** Estimates from linear CCREMs of cognitive function, 5-year birth cohort (*N* = 48918). **Figure S2.** Age, period, and cohort effects on cognitive function (5-year birth cohort). Note: Model 1 in Table S4 (left panel), Model 2 in Table S4 (right panel); the dotted line in the figure represents the estimated grand mean score of cognitive function. **Table S6.** Estimates from linear CCREMs of cognitive function, coding responses of “unable to answer” as wrong (*N* = 54789)856. **Figure S3.** Age, period, and cohort effects on cognitive function (coding responses of “unable to answer” as wrong). Note: Model 1 in Table S4 (left panel), Model 2 in Table S4 (right panel); the dotted line in the figure represents the estimated grand mean score of cognitive function. **Table S7.** Summary statistics of characteristics between excluded sample and study samples (unweighted).

## Data Availability

This study was based on the dataset from the CHARLS, and the CHARLS are freely available to all researchers. Information about the data source and access to data can be found at https://charls.charlsdata.com/pages/data/111/zh-cn.html.

## Abbreviations

HICs: High-income countries
LMICs: Low- and middle-income countries
APC: Age-period-cohort
CHARLS: China Health and Retirement Longitudinal Stud
TICS: Telephone interview of cognitive status
SES: Socioeconomic status
CCREM: Cross-classified random effects model
BIC: Bayesian Information Criterion
MCAR: Missing completely at random
CMD: Covariate-dependent missingness
HCAP: Harmonized Cognitive Assessment Protocol
