# Supplementary material for: New evidence of trends in cognitive function among middle-aged and older adults in China, 2011-2018: an age-period-cohort analysis
Source: BMC Geriatr. 2023 Aug 21;23:498. doi: 10.1186/s12877-023-04166-9 (PMC10440902; doi:10.1186/s12877-023-04166-9)
Supplement: Supplementary file 1 — Additional file 1: Table S1. Summary statistics of missingness in each cognitive domain by survey wave. Table S2. Summary statistics of missing data by survey wave. Table S3. Estimates of period and cohort effects from linear CCREMs of cognitive function (N = 48918). Table S4. Estimates from linear CCREMs of cognitive function, completely observed sample (N = 47571). Figure S1. Age, period, and cohort effects on cognitive function (completely observed sample). Note: Model 1 in Table S3 (left panel), Model 2 in Table S3 (right panel); the dotted line in the figure represents the estimated grand mean score of cognitive function. Table S5. Estimates from linear CCREMs of cognitive function, 5-year birth cohort (N = 48918). Figure S2. Age, period, and cohort effects on cognitive function (5-year birth cohort). Note: Model 1 in Table S4 (left panel), Model 2 in Table S4 (right panel); the dotted line in the figure represents the estimated grand mean score of cognitive function. Table S6. Estimates from linear CCREMs of cognitive function, coding responses of “unable to answer” as wrong (N = 54789)856. Figure S3. Age, period, and cohort effects on cognitive function (coding responses of “unable to answer” as wrong). Note: Model 1 in Table S4 (left panel), Model 2 in Table S4 (right panel); the dotted line in the figure represents the estimated grand mean score of cognitive function. Table S7. Summary statistics of characteristics between excluded sample and study samples (unweighted). [file 12877_2023_4166_MOESM1_ESM.docx]

# Additional file

**Table S1** Summary statistics of missingness in each cognitive domain by survey wave

| Domain | 2011 | 2013 | 2015 | 2018 | Total |
| --- | --- | --- | --- | --- | --- |
|  | n (%) | n (%) | n (%) | n (%) | N (%) |
| Episodic memory | 3570 (100) | 2828 (100) | 2076 (100) | 4076 (100) | 12550 (100) |
| Don’t know | 186 h5.2) | 0 (0) | 0 (0) | 0 (0) | 186 (1.5) |
| Refused | 60 (2.0) | 0 (0) | 397 (19.1) | 2423 (59.4) | 2880 (22.9) |
| Proxy | 1408 (47.7) | 461 (16.3) | 1202 (57.9) | 935 (22.9) | 4006 (31.9) |
| Missing responses | 1916 (64.9) | 2367 (83.7) | 477 (23.0) | 718 (17.6) | 5478 (43.6) |
| Orientation | 2952 (100) | 4248 (100) | 3582 (100) | 2596 (100) | 13378 (100) |
| Don’t know | 1366 (46.3) | 2457 (57.8) | 2213 (61.8) | 0 (0) | 6566 (49.1) |
| Refused | 17 (0.6) | 0 (0) | 16 (0.4) | 0 (0) | 33 (0.2) |
| Proxy | 1406 (47.6) | 460 (10.8) | 1191 (33.2) | 922 (35.5) | 3979 (29.7) |
| Missing responses | 163 (5.5) | 1331 (31.3) | 162 (4.5) | 712 (27.4) | 1838 (13.7) |
| Not assessed | 0 (0) | 0 (0) | 0 (0) | 962 (37.1) | 962 (7.2) |
| Attention | 3302 (100) | 3720 (100) | 3308 (100) | 6101 (100) | 16431 (100) |
| Don’t know | 1582 (47.9) | 1545 (41.5) | 1835 (55.5) | 2731 (44.8) | 7693 (46.8) |
| Refused | 143 (4.3) | 0 (0) | 94 (2.8) | 1724 (28.3) | 1961 (11.9) |
| Proxy | 1408 (42.6) | 461 (12.4) | 1204 (36.4) | 933 (15.3) | 4006 (24.4) |
| Missing responses | 169 (5.1) | 1714 (46.1) | 175 (5.3) | 713 (11.7) | 2771 (16.9) |
| Visuospatial ability | 1809 (100) | 2177 (100) | 1760 (100) | 4219 (100) | 9965 (100) |
| Don’t know | 94 (5.2) | 0 (0) | 171 (9.7) | 0 (0) | 265 (2.7) |
| Refused | 137 (7.6) | 0 (0) | 219 (12.4) | 0 (0) | 356 (3.6) |
| Proxy | 1408 (77.8) | 461 (21.2) | 1192 (67.7) | 932 (22.1) | 3993 (40.1) |
| Missing responses | 170 (9.4) | 1716 (78.8) | 178 (10.1) | 715 (16.9) | 2779 (27.9) |
| Not assessed | 0 (0) | 0 (0) | 0 (0) | 2572 (71.0) | 2572 (25.8) |

**Table S2** Summary statistics of missing data by survey wave

|  | 2011 | 2013 | 2015 | 2018 | Total |
| --- | --- | --- | --- | --- | --- |
| Characteristic | n = 11642 | n = 12145 | n = 14468 | n = 10633 | N = 48918 |
| Age | 0 (0) | 0 (0) | 0 (0) | 0 (0) | 0 (0) |
| Gender | 0 (0) | 0 (0) | 0 (0) | 0 (0) | 0 (0) |
| Marital status | 0 (0) | 3 (<0.1) | 1 (<0.1) | 0 (0) | 4 (<0.1) |
| Household registration | 0 (0) | 0 (0) | 0 (0) | 0 (0) | 0 (0) |
| Region | 0 (0) | 0 (0) | 0 (0) | 0 (0) | 0 (0) |
| Occupation | 159 (1.4) | 187 (1.5) | 274 (1.9) | 9 (<0.1) | 629 (1.3) |
| Education | 0 (0) | 0 (0) | 2 (<0.1) | 0 (0) | 2 (<0.1) |
| Drinking | 613 (5.3) | 38 (0.3) | 48 (0.3) | 9 (<0.1) | 708 (1.4) |
| Smoking | 1 (<0.1) | 1 (<0.1) | 2 (<0.1) | 8 (<0.1) | 12 (<0.1) |
| Social activities | 1 (<0.1) | 2 (<0.1) | 1 (<0.1) | 9 (<0.1) | 13 (<0.1) |
| Self-rated health | 4 (<0.1) | 7 (<0.1) | 8 (<0.1) | 12 (0.1) | 31 (<0.1) |

Note: Values are *n* (%)

**Table S3** Estimates of period and cohort effects from linear CCREMs of cognitive function (N = 48918)

| Random effects | Model 1 | Model 2 | Model 3 | Model 4 |
| --- | --- | --- | --- | --- |
|  | β (95% CI) | β (95% CI) | β (95% CI) | β (95% CI) |
| Period effects |  |  |  |  |
| 2011 | -0.22 (-0.52,0.07) | -0.20 (-0.47,0.07) | -0.01 (-0.15,0.13) | 0.02 (-0.12,0.14) |
| 2013 | 0.01 (-0.28,0.30) | 0.02 (-0.24,0.29) | 0.06 (-0.07,0.19) | 0.03 (-0.10,0.16) |
| 2015 | -0.19 (-0.47, 0.10) | -0.19 (-0.46,0.08) | **-0.17 (-0.30,-0.03)** | **-0.16 (-0.29,-0.04)** |
| 2018 | **0.40 (0.11, 0.70)** | **0.37 (0.10,0.64)** | 0.12 (-0.03,0.26) | 0.12 (-0.02,0.25) |
| Cohort effects |  |  |  |  |
| 1910-1930 | -0.25 (-0.65,0.15) | 0.02 (-0.37,0.41) | 0.50 (-0.01,1.01) | 0.37 (-0.10,0.84) |
| 1931-1933 | **0.59 (0.25,0.93)** | **0.46 (0.13,0.79)** | **0.69 (0.26,1.11)** | **0.50 (0.11,0.90)** |
| 1934-1936 | -0.19 (-0.48,0.10) | -0.18 (-0.47,0.10) | 0.07 (-0.31,0.45) | 0.02 (-0.32,0.37) |
| 1937-1939 | -0.08 (-0.34,0.18) | -0.19 (-0.44,0.07) | -0.05 (-0.39,0.29) | -0.06 (-0.37,0.25) |
| 1940-1942 | 0.12 (-0.11,0.36) | 0.17 (-0.06,0.39) | 0.08 (-0.24,0.39) | 0.08 (-0.21,0.36) |
| 1943-1945 | **0.46 (0.24,0.68)** | **0.47 (0.25,0.68)** | 0.25 (-0.05,0.55) | 0.26 (-0.01,0.52) |
| 1946-1948 | **0.26 (0.05,0.47)** | **0.28 (0.08,0.48)** | 0.10 (-0.18,0.38) | 0.14 (-0.12,0.40) |
| 1949-1951 | -0.05 (-0.25,0.15) | -0.07 (-0.26,0.13) | -0.07 (-0.35,0.21) | -0.03 (-0.28,0.22) |
| 1952-1954 | **-0.27 (-0.47,-0.07)** | **-0.30 (-0.50,-0.11)** | -0.27 (-0.56,0.01) | -0.22 (-0.47,0.03) |
| 1955-1957 | **-0.34 (-0.54,-0.14)** | **-0.37 (-0.57,-0.18)** | **-0.62 (-0.90,-0.33)** | **-0.54 (-0.80,-0.28)** |
| 1958-1960 | **-0.33 (-0.54,-0.11)** | **-0.37 (-0.58,-0.17)** | **-0.79 (-1.09,-0.48)** | **-0.72 (-1.00,-0.45)** |
| 1961-1963 | -0.22 (-0.45,0.00) | **-0.24 (-0.47,-0.02)** | **-0.68 (-1.01,-0.36)** | **-0.61 (-0.91,-0.32)** |
| 1964-1966 | -0.09 (-0.34,0.16) | -0.08 (-0.32,0.17) | **-0.38 (-0.73,-0.03)** | **-0.33 (-0.65,-0.01)** |
| 1967-1969 | 0.07 (-0.22,0.35) | 0.09 (-0.19,0.37) | 0.31 (-0.08,0.70) | 0.30 (-0.05,0.66) |
| 1970-1973 | 0.31 (-0.22,0.65) | 0.32 (0.00,0.64) | **0.86 (0.43,1.30)** | **0.83 (0.43,1.23)** |

Note: Values in bold indicate significance below the level of 0.05

**Table S4** Estimates from linear CCREMs of cognitive function, completely observed sample (N = 47571)

| Fixed effects | Model 1 | Model 2 |
| --- | --- | --- |
|  | β (95% CI) | β (95% CI) |
| Intercept | 12.88 (12.54,13.23) | 13.29 (10.98,11.64) |
| Age/10 | -0.98 (-1.10,-0.85) | -0.60 (-0.75,-0.45) |
| Age^2^/100 | -0.15 (-0.20,-0.09) | -0.25 (-0.30,-0.20) |
| Women | / | -0.22 (-0.30,-0.15) |
| Having spouse | / | 0.36 (0.29,0.43) |
| Urban | / | 0.69 (0.64,0.75) |
| Region | / |  |
| East |  | 1(Reference) |
| Central |  | -0.22 (-0.28,-0.17) |
| West |  | -0.49 (-0.55,-0.43) |
| Education | / |  |
| ≥middle school |  | 1(Reference) |
| <middle school |  | -1.51 (-1.57,-1.45) |
| Illiterate |  | -4.23 (-4.31,-4.14) |
| Occupation | / |  |
| Nonagricultural job |  | 1(Reference) |
| Nonworking |  | -0.08 (-0.15,-0.01) |
| Agricultural job |  | -0.44 (-0.51,-0.37) |
| Drinking | / |  |
| Nondrinker |  | -0.14 (-0.19,-0.08) |
| Light/moderate |  | 1(Reference) |
| Heavy |  | -0.65 (-0.87,-0.43) |
| Smoking | / |  |
| Nonsmoker |  | 1(Reference) |
| Light/moderate |  | -0.26 (-0.34,-0.19) |
| Heavy |  | -0.48 (-0.58,-0.39) |
| Participating in social activities | / | 0.57 (0.51,0.62) |
| SRH | / | 0.24 (0.22,0.27) |
| Random effects (variance components) | σ (95% CI) | σ (95% CI) |
| Period | 0.091 (-0.057,0.239) | 0.019 (-0.012,0.050) |
| Cohort | 0.088 (0.013,0.163) | 0.199 (0.036,0.362) |
| BIC | 254099 | 239267 |

Note: Random effect coefficients are omitted in the interest of space, sampling weights were used in the model; *CCREMs* Cross-classified random-effects models, *CHARLS* China Health and Retirement Longitudinal Study, *SRH* Self-rated health, *BIC* Bayesian Information Criterion

**Figure S1** Age, period, and cohort effects on cognitive function (completely observed sample)

Note: Model 1 in Table S3 (left panel), Model 2 in Table S3 (right panel); the dotted line in the figure represents the estimated grand mean score of cognitive function

**Table S5** Estimates from linear CCREMs of cognitive function, 5-year birth cohort (N = 48918)

| Fixed effects | Model 1 | Model 2 |
| --- | --- | --- |
|  | β (95% CI) | β (95% CI) |
| Intercept | 12.70 (12.34,13.06) | 13.07 (12.78,13.35) |
| Age/10 | -0.81 (-0.95,-0.66) | -0.53 (-0.65,-0.41) |
| Age^2^/100 | -0.13 (-0.18,-0.07) | -0.20 (-0.25,-0.16) |
| Women | / | -0.25 (-0.32,-0.17) |
| Having spouse | / | 0.37 (0.31,0.44) |
| Urban | / | 0.73 (0.67,0.78) |
| Region | / |  |
| East |  | 1(Reference) |
| Central |  | -0.23 (-0.29,-0.17) |
| West |  | -0.49 (-0.55,-0.43) |
| Education | / |  |
| ≥middle school |  | 1(Reference) |
| <middle school |  | -1.45 (-1.51,-1.40) |
| Illiterate |  | -4.17 (-4.25,-4.08) |
| Occupation | / |  |
| Nonagricultural job |  | 1(Reference) |
| Nonworking |  | -0.10 (-0.17,-0.03) |
| Agricultural job |  | -0.46 (-0.53,-0.39) |
| Drinking | / |  |
| Nondrinker |  | -0.12 (-0.18,-0.06) |
| Light/moderate |  | 1(Reference) |
| Heavy |  | -0.66 (-0.88,-0.44) |
| Smoking | / |  |
| Nonsmoker |  | 1(Reference) |
| Light/moderate |  | -0.28 (-0.36,-0.21) |
| Heavy |  | -0.50 (-0.59,-0.41) |
| Participating in social activities | / | 0.58 (0.53,0.63) |
| SRH | / | 0.24 (0.22,0.27) |
| Random effects (variance components) | σ (95% CI) | σ (95% CI) |
| Period | 0.067 (-0.044,0.178) | 0.015 (-0.010,0.040) |
| Cohort | 0.121 (-0.078,0.321) | 0.085 (-0.002,0.173) |
| BIC | 261310 | 246191 |

Note: Random effect coefficients are omitted in the interest of space, sampling weights were used in the model; *CCREMs* Cross-classified random-effects models, *CHARLS* China Health and Retirement Longitudinal Study, *SRH* Self-rated health, *BIC* Bayesian Information Criterion

**Figure S2** Age, period, and cohort effects on cognitive function (5-year birth cohort)

Note: Model 1 in Table S4 (left panel), Model 2 in Table S4 (right panel); the dotted line in the figure represents the estimated grand mean score of cognitive function

**Table S6** Estimates from linear CCREMs of cognitive function, coding responses of “unable to answer” as wrong (N = 54789)856

| Fixed effects | Model 1 | Model 2 |
| --- | --- | --- |
|  | β (95% CI) | β (95% CI) |
| Intercept | 11.95 (11.33,12.56) | 13.13 (12.65,13.62) |
| Age/10 | -1.23 (-1.36,-1.11) | -0.70 (-0.88,-0.52) |
| Age^2^/100 | -0.22 (-0.28,-0.16) | -0.32 (-0.38,-0.27) |
| Women | / | -0.45 (-0.53,-0.37) |
| Having spouse | / | 0.42 (0.35,0.49) |
| Urban | / | 0.80 (0.74,0.85) |
| Region | / |  |
| East |  | 1(Reference) |
| Central |  | -0.33 (-0.40,-0.27) |
| West |  | -0.72 (-0.79,-0.66) |
| Education | / |  |
| ≥middle school |  | 1(Reference) |
| <middle school |  | -1.71 (-1.77,-1.65) |
| Illiterate |  | -5.25 (-5.33,-5.16) |
| Occupation | / |  |
| Nonagricultural job |  | 1(Reference) |
| Nonworking |  | -0.21 (-0.29,-0.14) |
| Agricultural job |  | -0.48 (-0.55,-0.41) |
| Drinking | / |  |
| Nondrinker |  | -0.10 (-0.17,-0.04) |
| Light/moderate |  | 1(Reference) |
| Heavy |  | -0.63 (-0.85,-0.41) |
| Smoking | / |  |
| Nonsmoker |  | 1(Reference) |
| Light/moderate |  | -0.21 (-0.29,-0.13) |
| Heavy |  | -0.34 (-0.44,-0.24) |
| Participating in social activities | / | 0.67 (0.62,0.72) |
| SRH | / | 0.27 (0.24,0.30) |
| Random effects (variance components) | σ (95% CI) | σ (95% CI) |
| Period | 0.366 (-0.044,0.178) | 0.130 (-0.079,0.339) |
| Cohort | 0.121 (-0.078,0.321) | 0.290 (0.049,0.532) |
| BIC | 301976 | 279564 |

Note: Random effect coefficients are omitted in the interest of space, sampling weights were used in the model; *CCREMs* Cross-classified random-effects models, *CHARLS* China Health and Retirement Longitudinal Study, *SRH* Self-rated health, *BIC* Bayesian Information Criterion

**Figure S3** Age, period, and cohort effects on cognitive function (coding responses of “unable to answer” as wrong)

Note: Model 1 in Table S4 (left panel), Model 2 in Table S4 (right panel); the dotted line in the figure represents the estimated grand mean score of cognitive function

**Table S7** Summary statistics of characteristics between excluded sample and study samples (unweighted)

| Characteristics | (1) Excluded sample  N = 25620^a^ | | (2) Study sample  N = 48918 | Diff (1) vs. (2) | Effect size^c^ |
| --- | --- | --- | --- | --- | --- |
|  | n | M (SD) / % | M (SD) / % | *P*^b^ | $\phi_{c}$ / $\eta^{2}$ |
| Age | 25160 | 61.8 (10.3) | 59.0 (9.1) | <.001 | 0.031 |
| Gender | 25612 |  |  | <.001 | 0.043 |
| Male | 12386 | 48.4 | 52.9 |  |  |
| Women | 13226 | 51.6 | 47.2 |  |  |
| Marital status | 25531 |  |  | <.001 | 0.036 |
| Having spouse | 20746 | 81.3 | 84.1 |  |  |
| Alone | 4785 | 18.7 | 15.9 |  |  |
| Household registration | 25620 |  |  | 0.002 | 0.011 |
| Rural | 14838 | 57.9 | 56.7 |  |  |
| Urban | 10782 | 42.1 | 43.3 |  |  |
| Region | 25620 |  |  | 0.004 | 0.012 |
| West | 8210 | 31.7 | 30.6 |  |  |
| Central | 8591 | 33.5 | 33.7 |  |  |
| East | 8909 | 34.8 | 35.7 |  |  |
| Education | 25584 |  |  | <.001 | 0.142 |
| Illiterate | 6875 | 26.9 | 15.2 |  |  |
| <middle school | 10299 | 40.3 | 45.0 |  |  |
| ≥middle school | 8410 | 32.9 | 39.9 |  |  |
| Occupation | 24631 |  |  | <.001 | 0.016 |
| Nonworking | 8418 | 34.2 | 32.8 |  |  |
| Agricultural job | 8860 | 36.0 | 35.9 |  |  |
| Nonagricultural job | 7353 | 29.9 | 31.3 |  |  |
| Drinking | 24411 |  |  | <.001 | 0.074 |
| Nondrinker | 17150 | 70.3 | 62.9 |  |  |
| Light/moderate | 6893 | 28.2 | 35.6 |  |  |
| Heavy | 368 | 1.5 | 1.5 |  |  |
| Smoking | 25062 |  |  | <.001 | 0.080 |
| Nonsmoker | 14704 | 58.7 | 54.7 |  |  |
| Light/moderate | 8244 | 32.9 | 31.4 |  |  |
| Heavy | 2114 | 8.44 | 14.0 |  |  |
| Social activities | 21073 |  |  | <.001 | 0.038 |
| Participating | 8365 | 43.4 | 52.4 |  |  |
| Not participating | 10908 | 56.6 | 47.6 |  |  |
| Self-rated health | 24076 | 2.9 (1.0) | 3.1 (1.0) | <.001 | 0.009 |

Note: ^a^The excluded sample is respondents with missingness on age/cognitive tests/survey weight among those aged 45 years or older, so the sample size is calculated as 74538-48918=25620

^b^The reported *P* value is for a $\chi^{2}$ test or One-Way ANOVA for significant differences in proportion or mean between excluded sample and study sample

^c^The effect size is $\phi_{c}$ (Cramér’s V) for $\chi^{2}$ test and $\eta^{2}$ for ANOVA
